# Comparative chemical and antimicrobial evaluation of the essential oils from Callistemon and podocarpus species supported by *in-silico* molecular simulations against bacterial LacY protease

**DOI:** 10.1186/s12906-025-04826-w

**Published:** 2025-04-21

**Authors:** Heba A. M. Ezzat, Nermin A. Younis, Mai M. Zafer, Amel M. Kamal, Mohamed I. S. Abdelhady, Mohamed S. Mady

**Affiliations:** 1 Department of Pharmacognosy, Faculty of Pharmacy, Ahram Canadian University, Giza, Egypt; 2 Department of Pharmacognosy, Faculty of Pharmacy, Helwan University, Ein Helwan, Cairo, 11795 Egypt; 3 Department of Microbiology and Immunology, Faculty of Pharmacy, Ahram Canadian University, Cairo, Egypt

**Keywords:** Antimicrobial, Essential oil, Molecular docking, Lactose permease, Efflux pump

## Abstract

**Background:**

Myrtaceae and Podocarpaceae botanical families include several aromatic species that have been proven to have diverse pharmacological potential, especially antimicrobial effects. Additionally, plants of both families were reported for their benefits in traditional medicine. The current study demonstrated the chemical profile, antimicrobial of four investigated plant species (*C. subulatus, C. rigidus, P. gracilior,* and* P. elongatus*) leaves cultivated in the same place in Egypt and propose in-silico modeling for the antibacterial mechanistic action.

**Method:**

The essential oils samples were prepared via hydrodistillation and headspace extraction protocol and GC analysis was conducted to obtain a comparative chemical profile. The antimicrobial activity of the obtained hydrodistillation essential oil samples was screened via agar diffusion, and the MIC was calculated via broth microdilution assays. An in silico molecular docking study was performed to investigate the inhibition of the LacY protease efflux pump.

**Results:**

GC results revealed that the percentage of oxygenated monoterpenes was highest in the oil samples from Callistemon species (60.38 and 82.68%). In contrast, sesquiterpene hydrocarbons constituted the highest percentage of volatile classes in the oil samples from Podocarpus species (57.37 and 43.16%). *C. rigidus*-EO shows significant inhibitory activity against Gram-negative and Gram-positive pathogens, especially *E. coli* and *S. viridans,* with a calculated MIC of 0.878 ml, whereas *P. elongatus* EO shows notable activity against *coagulase-negative Staphylococcus* and the activity was comparable to that of the positive control antibiotics used (ciprofloxacin & doxycycline). Ultimately, an in silico molecular docking study on the binding site of the LacY protease enzyme revealed a significant binding affinity of the major docked volatile constituents.

**Conclusion:**

The plant species investigated are considered a vital source of safe antimicrobial volatile constituents that are recommended as bioactive entities for controlling microbial infection topically or systemically. The proposed mechanistic action encourages further modification of the major EOs chemical structure by adding more polar substitutions to improve the binding affinity and produce more active semisynthetic analogues.

**Supplementary Information:**

The online version contains supplementary material available at 10.1186/s12906-025-04826-w.

## Introduction

Plants are considered a treasure filled with different libraries of chemical entities that have several benefits for humans, ranging from nutritional to medicinal significance. The secondary metabolites produced by plants are a product of physiological functions inside the plant cell or as a response to external stimulants as a defense mechanism [1]. Essential oils (EOs) The odoriferous components produced by plant organs, which are liquid and volatile with different scents according to their composition. Several factors influence the number of EOs extracted, such as climate, geographical aspects, collection time, and extraction procedures [2, 3]. The most common techniques used in the extraction of EOs are hydrodistillation, effleurage, steam distillation, solvent extraction, supercritical fluid, and microwave-assisted extraction [4]. The major constituent of EOs is terpenes. The building unit of terpenes is an isoprene unit (C_5_H_8_). The number of isoprene units results in different classes of terpenes and volatile oils [4]. The importance of EOs ranges from medicinal benefits to the cosmetic industry, as they are used as raw materials in the production of perfumes, cosmetics, and aromatherapy [5, 6]. With respect to EO samples, modern methods have been established to obtain oil samples with high purity to overcome the limitations of traditional methods and enhance extraction outcomes. Headspace microextraction is a new strategy for microextraction EOs directly from fresh plant samples and offers a rapid and efficient extraction method, especially when the sample amount is limited [6, 7]. Therefore, the optimal extraction technique is selected according to the desired EO quality and constituents, which ultimately support the desired pharmacological benefits [8].

Increasing microbial resistance is urgently needed for the continuous search for new antimicrobial agents with notable effectiveness [9]. The global prevalence of antimicrobial-resistant bacteria has reached alarming levels, posing a significant threat to public health on a global scale, such as during a silent pandemic. This urgent situation necessitates immediate intervention. Infections caused by antimicrobial-resistant bacteria present a serious challenge, as therapeutic options are limited, leading to substantial morbidity, mortality, and a significant economic burden. The scarcity of new and innovative antimicrobial agents to address life-threatening infections caused by resistant pathogens contrasts completely with the growing demand for effective treatments [10]. For decades, plants have offered the opportunity for the discovery of many structurally diverse phytochemicals. The antimicrobial potential of EOs has been well reported, and reports have correlated their efficacy with the chemical nature of the EOs on the basis of their hydrophobic nature, which is mainly hydrocarbons that can sometimes be oxygenated, leading to an increase in the potential for strong hydrophobic interactions with protein-substrate binding sites as a mechanism of action and facilitating membrane transport and permeability [11, 12].

Among plant families, the family Podocarpaceae is well known to include several aromatic trees and shrubs. The family Podocarpaceae consists of evergreen trees or shrubs, including approximately 20 genera and more than 200 species. Warm regions are considered the native sources of these plants, especially in the Southern Hemisphere, and some of these plants are grown as ornamental plants [13]. *Podocarpus* is a genus that comprises approximately 100 species and is widely spread throughout Mexico, South Africa, southern China, and southern Japan [14]. A wide range of phytochemicals, including terpenes [15–23], essential oils [24], phenolics [16, 20, 25], and sterols [26], have been isolated and identified from the genus Podocarpus. Species of this genus were reported to possess several pharmacological importance, such as anti-inflammatory, antioxidant [27, 28], antiseptic, for the treatment of chest complaints [29], carminative, antiulcer, stomachic [29, 30], and cytotoxic effects [20, 31]. *Podocarpus gracilior* and *Podocarpus elongatus* were cultivated *Podocarpus* species in Egypt. *P. gracilior* (Pilger) is a tree that natively grows in Central and East Africa and is known as African Fern Pine [16, 32]. Several reports have highlighted the pharmacological importance of *P. gracilior,* such as the anticancer and cytotoxic activities of its ethanol extract [33, 34]. *P. elongatus* Aiton L'Hér. ex Pers is a tree that natively grows in South Africa and is known as de River yellowwood. Additionally, several reports have indicated the medicinal importance of *P. elongatus,* such as its crude extracts, which exhibit broad-spectrum antimicrobial activity [23, 35].

The family Myrtaceae is a well-known plant family that includes approximately 140 genera, including many important trees and shrubs. *Callistemon* is one of the Myrtle family members and comprises approximately forty species native to Australia [36]. *Callistemon* species are well known for their use in folk medicine, as they have valuable medicinal value. These species have been used for the treatment of lung diseases, skin problems, and GIT manifestations [37]. *C. sabulatus* Cheel and *C. rigidus* R.Br have been reported several times for their pharmacological benefits in terms of their phenolic content and essential oils [36, 38].

Ongoing research has focused on the comparative exploration of essential oil (EO) chemical profiles derived from four plant species belonging to the Callistemon and Podocarpus genera in Egypt. Identification of essential oil constituents is performed through gas chromatography (GC) analysis. This study aims to assess and compare the chemical compositions and major components of essential oils extracted using two different extraction methods, hydodistillation as example of conventional old extraction technique and headspace as example of advanced extraction method using two species of two different plant genera growing in the same place and collected at the same seasonal period. Additionally, research has focused on evaluating the antimicrobial potential of the extracted essential oils using conventional hydrodistillation technique, since it is the most available method of extraction. Finally, in order to gain more insight into the potential impact of the identified oils on a membrane transporter, in silico molecular docking was employed as a supplementary analytical approach.

## Materials and methods

### Plant material

The aerial parts of *Callistemon subulatus* Cheel, *Callistemon rigidus* R.Br., *Podocarpous gracilior* Pilg., and *Podocarpous elongatus* Aiton L'Hér. ex Pers were collected from Orman Garden, El-Giza, Egypt (February 2022), according to the garden guidelines, and the botanical sample collection rules stated in Egypt. Taxonomic authentication of the samples was performed by Dr. Trease Labib, a plant taxonomist at Mazhar Garden, El-Giza, Egypt. A voucher sample was deposited at the official Herbarium of the Department of Pharmacognosy, Faculty of Pharmacy, Helwan University, Cairo, Egypt, under official serial numbers 39*Pgr*1/2024, 39*Pel*2/2024, 16*Csa*2/2024 and 16*Cri*4/2024 for *P. gracilior* and* P. elongatus, C. subulatus and C. rigidus,* respectively.

### Essential oil extraction

#### Hydrodistillation technique

Fresh leaves (750 g) of the mentioned species (Plant material section) were subjected separately to the traditional hydrodistillation method following a previously reported procedure [38]. The leaves were comminuted, distilled water was added until they covered the surface, and the EO hydrodistillation method was performed via a Clevenger apparatus. The distillate was allowed to cool at room temperature, the essential oil was allowed to separate from the water, and each essential oil sample was weighed on an analytical scale (3.75 ml 0.83% v/w, 2.5 ml 0.559% v/w, 0.25 ml 0.025 and 0.2 ml 0.02% v/w) from *C. rigidus, C. subulatus, P. gracilior, and P. elongatus,* respectively. The obtained EOs were passed over anhydrous Na_2_SO_4_ for dehydration and then stored in amber-colored vials at 4 °C for GC/MS analysis and biological activity investigation.

#### Head-space solid-phase microextraction

Headspace solid-phase (HS) microextraction of the EOs from the collected fresh samples was carried out according to the procedure reported in the literature [39]. Approximately 2 g of collected fresh leaves was placed separately into a glass vial (5 mL). The vial's optimum temperature was maintained at 60–70 °C to create a saturated headspace of the solid samples in the vial with the EO vapor. The solid-phase microextraction syringe was mounted in the headspace, and then, the EO vapors were absorbed by the needle of the syringe (silica phase). The saturated silica fiber of the syringe was fixed to the GC/MS injection unit for the estimation of the EO components.

### Gas chromatography analysis of the EOs

For estimation of the obtained EO components, the mass spectrum was generated via a Shimadzu GC/MS-QP2010 system (Kyoto, Japan) equipped with a mass spectrometer (quadrupole) (Shimadzu Corporation, Kyoto, Japan). GC separation was performed on a column [Rtx- (30 m × 0.25 mm i.d. × 0.25-μm film thickness] 5MS column from Restek, United States. All the obtained EOs were subsequently estimated according to the same conditions and as reported previously [38, 40].

### Identification of essential oil components

The obtained oil samples were estimated individually via GC analysis. The identification of the EO constituents was determined tentatively on the basis of the component retention indices (RIs) in comparison with those of authentic *n*-alkanes (C_8_–C_28_); then, NIST and Wiley mass spectra library databases and previously reported data were used for comparison of their mass spectra for more confirmation (similarity index > 90%) [41, 42]. The retention indices were obtained by using the GC/MS solution program.

### Antimicrobial activity of the obtained EOs

#### Microbes, positive controls, and culture media

In this study, the following microorganism strains were used to assess the antibacterial and antifungal activities of the essential oils. Gram-positive bacteria *such as methicillin-resistant Staphylococcus aureus* (MRSA), *Streptococcus viridans, Enterococcus faecalis, coagulase-negative Staphylococcus (*CoNS), Gram-negative bacteria *Escherichia coli*, and *Candida albicans* fungi. All of these are clinical isolates that were recovered from hospitalized patients and those strains were multidrug resistant to commonly used antibiotics. The isolates were cultured on Mueller–Hinton broth and streaked on blood agar. The *Candida albicans* isolate was maintained on a potato dextrose agar (PDA) slant. All the isolates were identified via VITEK II (bioMerieux, Marcy l’E´toile, France). The bacterial suspensions were adjusted to the desired concentration equivalent to that of a 0.5 McFarland standard and incubated overnight under aerobic conditions at 37°C.

#### Agar well diffusion assay

Das et al. (2013) reported a protocol in which the agar well diffusion method was used to evaluate the antibacterial activity of essential oils [43]. Water and Tween 80 (less than 10%) were added in a 1:1 ratio (v/v) to the oil. The mixture was mixed and then subjected to 30 min of sonication and vortex homogenization for 8 min to obtain a stable essential oil emulsion, which was subsequently stored at 4^○^C for further use. The culture plates were allowed to solidify, and then, a volume of 100 μL (1.5 × 10^8^ CFU/mL) of fresh inoculum of the tested bacteria (MRSA, CoNS, *S. viridans*, *E. faecalis, E. coli)* was streaked onto Mueller–Hinton agar. The plates were subsequently punched with a sterile cork borer, and the opened wells were inoculated with 50 µL of the tested essential oil mixture. The standard antibiotics doxycycline (30 µg) and ciprofloxacin (5 µg) were used as controls. All the plates were incubated at 37°C for 24 h. For *C. albicans, the* resulting inhibition zones (ZOIs) were measured after the plates were incubated at 28°C for 24 h. The experiment was performed in triplicate, and the average inhibition zone size was determined.

#### Minimum Inhibitory Concentration (MIC) determination

The MIC was tested via broth microdilution via a resazurin microtiter plate-based antibacterial assay [44]. Stable essential oil emulsions were used as described above. In a 96-well microtiter plate, 100 µL of sterile nutrient broth and 100 µL of stable essential oil emulsion were added to the first well. Eleven-fold serial dilutions were performed by transferring 100 mL from well to well (in rows). To each well, 10 µL of inoculum (1.5 × 108 CFU/mL) was added. The concentrations reached ranged from 225 µL to 0.878 µL EO/mL. The microplates were incubated for 18–24 h at 37^○^C. Thirty microliters of resazurin aqueous solution were added to each well. The microtiter plates were incubated at 37 °C for 2 h. The concentration at which the blue color did not turn pink was considered the MIC. Three replicates were performed for each essential oil.

## Molecular docking studies

### Preparation of protein structure

Lactose permease enzyme (LacY), a membrane protein that is a member of the facilitator family of transporters, was selected from the Protein Data Bank (www.rscb.org). This enzyme (PDB ID: 1PV7 [45]), which consists of C-terminal and N-terminal domains, has 6 symmetrically positioned transmembrane helices. This transmembrane has a large hydrophilic cavity to the inside that opens toward the cytoplasm, indicating that the membrane transporter has an inward facing conformation. The Molecular Operating Environment 2022 (MOE 2022) program was employed to decrease the tension of the crystal structure and to prepare a stable protein structure. MOE was used to delete the hydrogens from the macromolecules and again added to make the enzyme suitable as a protonated macromolecule at a convenient pH and temperature. Energy minimization through the omission of water molecules and ligands. The whole protein was scanned, and optimization steps were performed to allocate the active sites on the protein.

### Ligand preparation

The 2D structures of the ligand molecules were sketched via ChemDraw 19.1 (Perkin Elmer Informatics) software, and a database was generated, transferred to MOE software, and converted into a 3D structure. Then, the file was saved as a mdb file extension. Here, ten volatile oil molecules (Table 4) were optimized and introduced into the ligand dataset.

### Protein–ligand modeling simulation

The MOE 2020 program was used to create the design, locate the active site, and generate the modeling files. Here, the ligand-binding site is in the hydrophilic cavity at the same distance from both sides of the membrane. The modeling study was adjusted to yield 30 poses of the most stable conformers for each ligand with different torsion angles. The data generated from the modeling study were interpreted according to the S (Score) value, which indicates the binding energy and the possible binding affinity.

## Results and discussion

Essential oils (EOs) are mixtures of volatile components that are characterized by their diverse chemical entity, notable aroma, and valuable pharmacological potential [46]. In general, each aromatic species yields a “fingerprint” of EOs, which varies according to the extracted organ and its geographical origin. These parameters usually affect the biological potential of EOs in either a synergistic, positive, or antagonistic way [47]. Another parameter that affects the overall potential and composition of EOs is the method of extraction [48]. First, the hydrodistillation (HD) technique is the most frequently employed method, but the resulting EOs can fluctuate in response to several factors, such as polymerization, saponification, and/or isomerization. Accordingly, in this work, we report the extraction of the EOs of the aerial parts of four different species growing in Egypt (*C. subulatus, C. rigidus, P. gracilior,* and* P. elongatus*) via HD and HS methods to investigate the differences in their analysis outcomes in terms of chemical composition and antimicrobial potential.

The extraction techniques affect the composition of the obtained EO in many ways. For example, regarding the EOs obtained from *Callistemon* species, a total of 22 and 9 volatile constituents were estimated in HD- and HS-derived oils, respectively, from *C. subulatus*, whereas a total of 14 and 11 volatile constituents were estimated in HD- and HS-derived oils, respectively, from *C. rigidus* (Table 1, Supplementary Figure S1–S2). On the other hand, in the EOs obtained from *Podocarpous* species, a total of 32 and 7 volatile constituents were estimated in HD- and HS-derived oils, respectively, from *P. gracilior*, whereas a total of 17 and 7 volatile constituents were estimated in HD- and HS-derived oils, respectively, from *P. elongatus* (Table 2, Supplementary Figure S3–S4). Moreover, variability in the percentage of the EO chemical class of the identified compounds was also observed.

**Table 1:** Percent concentration (%) of the volatile content estimated in the EOs of *C. subulata* and *C. rigidus* aerial parts extracted using hydro distillation (HD), and headspace solid-phase micro-sampling

No	Identified Compounds	MF	M. wt	RI_exp_	RI _lit_	*C. subulatus*		*C. rigidus*	
						**HD Area%**	**HS Area%**	**HD Area%**	**HS Area%**
1	*α*-Thujene	C_10_H_16_	136	909	909	1.50	2.06		0.06
2	*α*-Pinene	C_10_H_16_	136	918	918	**22.42**	**27.96**	3.64	0.86
3	Sabinen	C_10_H_16_	136	967	966			0.43	0.36
4	*β*-Pinene	C_10_H_16_	136	971	980	1.25	0.64	1.70	0.50
5	*β*-Myrcene	C_10_H_16_	136	989	987	0.73		2.65	1.14
6	Pseudolimonene	C_10_H_16_	136	1000	1000			0.34	
7	*α* -Phyllandrene	C_10_H_16_	136	1002	1001	7.61	3.84		
8	*α* -Terpinene	C_10_H_16_	136	1049	1049	0.29		0.65	0.09
9	Cymene	C_10_H_14_	134	1017	1015	5.56	4.46	1.37	0.15
10	Eucalyptol	C_10_H_18_O	154	1026	1022	**38.29**	**59.53**	**69.93**	**93.32**
11	trans-* β* -Ocimene	C_10_H_16_	136	1033	1033	0.73			
12	*γ*-Terpinene	C_10_H_16_	136	1055	1049	1.19	0.66	0.66	0.26
13	*α* -Terpinoline	C_10_H_16_	136	1084	1084	0.77	0.32		
14	Linalool	C_10_H_18_O	154	1091	1090	1.34	0.53	2.80	1.72
15	Pinocarveol	C_10_H_16_O	152	11,225	1130	0.45			
16	Borneol	C_10_H_18_O	154	1152	1151	0.36			
17	α-Terpineol	C_10_H_18_O	154	1176	1183	7.00		9.24	1
18	Geraniol	C_10_H_18_O	154	1241	1241	0.30		0.39	
19	Eugenol	C_10_H_12_O_2_	164	1333	1333	0.25			
20	Cinerolon	C_10_H_12_O_2_	164	1496	1496	0.62			
21	Geranyl acetate	C_12_H_20_O_2_	196	1366	1366	0.33			
22	Spathulenol	C_15_H_24_O	220	1562	1562	0.22			
23	Viridiflorol	C_15_H_26_O	222	1567	1567	0.96			
24	Globulol	C_15_H_26_O	222	1571	1571			1.17	
25	Rosifoliol	C_15_H_26_O	222	1607	1607	0.52		0.34	
**Monoterpene hydrocarbons**						**41.28**	**39.62**	**11.44**	**3.42**
**Oxygenated Monoterpene**						**51**	**60.38**	**82.68**	**96.04**
**Sesquiterpene hydrocarbons**									
**Oxygenated Sesquiterpene**						**1.70**		**1.51**	
**Others**						**4.65**		**4.37**	**0.54**
**Total identified**						**98.63**	**100**	**100**	**100**
**Unidentified**						**1.37**			

*MF* Molecular formula, *M. wt.* Molecular weight, *BP* Base peak, *RI*_*Exp*_ experimental refractive index, *RI*_*Lit*_ reference refractive index

**Table 2:** Percent concentration (%) of the volatile content estimated in the EOs of *P. gracilior* and *P. elongatus* aerial parts extracted using hydro distillation (HD), and headspace solid-phase micro-sampling

No	Identified Compounds	MF	M. wt	RI_exp_	RI _lit_	*P. gracilior*		*P. elongatus*	
						**HD Area%**	**HS Area%**	**HD Area%**	**HS Area%**
1	*α*-Pinene	C_10_H_16_	136	918	918	**12.2**	**82.01**	0.98	4.54
2	*β*-Pinene	C_10_H_16_	136	971	980	0.83			0.73
3	Myrcene	C_10_H_16_	136	989	987	4.34	**13.29**	4.64	**79.05**
4	*β*-Phellandrene	C_10_H_16_	136	1002	1001	0.49	0.64		
5	m-Mentha-6,8-diene (Sylvestrene)	C_10_H_16_	136	1017	1017	0.19	0.22	1.54	12.48
6	*β*-Ocimene	C_10_H_16_	136	1037	1036	0.09			0.73
7	Terpinolene	C_10_H_16_	136	1075	1074	0.09			
8	*α* -Terpineol	C_10_H_18_O	154	1167	1170	0.16			
9	*α* -Cubebene	C_15_H_24_	204	1343	1344	1.83			
10	Copaene	C_15_H_24_	204	1369	1367	6.22	0.55		
11	*β*-caryophyllene	C_15_H_24_	204	1419	1419	**16.14**	0.79	3.91	
12	trans-*β*-copaene	C_15_H_25_	205	1417	1415			0.89	
13	Aromadendrene	C_15_H_24_	204	1428	1425			0.58	
14	Humulene	C_15_H_24_	204	1441	1441	4.04		1.13	
15	cadina-3,5-diene	C_15_H_24_	204	1449	1445	0.30			
16	*γ*-Muurolene	C_15_H_24_	204	1454	1453	0.21		1.93	
17	Germacrene D	C_15_H_24_	204	1469	1469	**15.66**	0.55	**12.83**	1.38
18	cis-Muurola-4(15),5-diene	C_15_H_24_	204	1477	1476	0.77			
19	Bicyclogermacrene	C_15_H_24_	204	1482	1481	1.05		**17.3**	0.72
20	*α* -Muurolene	C_15_H_24_	204	1486	1485	4.29			
21	*γ* -Amorphene	C_15_H_24_	204	1497	1495	0.67		1.19	
22	*α* -Cadinene	C_15_H_24_	204	1506	1505			3.40	
23	Cadina-3,9-diene	C_15_H_24_	204	1508	1508	5.76			
24	Cubenene	C_15_H_24_	204	1516	1516	0.17			
25	*α* -Bisabolene	C_15_H_24_	204	1526	1525	0.16			
26	Nerolidyl acetate	C_17_H_28_O_2_	264	1542		0.27			
27	Spathulenol	C_15_H_24_O	220	1562	1562	0.46		6.12	
28	Globulol	C_15_H_26_O	222	1571	1571			2.58	
29	gleenol	C_15_H_26_O	222	1568	1567	0.17			
30	Viridiflorol	C_15_H_26_O	222	1567	1567			2.25	
31	Cedrol	C_15_H_26_O	222	1589	1590	0.31			
32	Junenol	C_15_H_26_O	222	1596	1595	0.21			
33	Di-epi-1,10-cubenol	C_15_H_26_O	222	1605	1607	1.14			
34	tau-Muurolol	C_15_H_26_O	222	1617	1617	4.94		4.63	
35	*α*-Cadinol	C_15_H_26_O	222	1629	1630	3.62		4.46	
37	Arctiol	C_15_H_26_O_2_	238	1643	1640	0.27			
**Monoterpene hydrocarbons**						**18.23**	**96.16**	**7.16**	**97.53**
**Oxygenated Monoterpene**						**0.16**			
**Sesquiterpene hydrocarbons**						**57.37**	**1.89**	**43.16**	**2.1**
**Oxygenated Sesquiterpene**						**13.23**		**22.4**	
**Others**						**10.41**	**1.95**	**26.4**	**0.37**
**Total identified**						**99.4**	**100**	**99.12**	**100**
**Unidentified**						**0.33**		**0.87**	

*MF* Molecular formula, *M. wt.* Molecular weight, *BP* Base peak, *RI*_*Exp*_ experimental refractive index, *RI*_*Lit*_ Reference refractive index

Based on the GC results for the EOS samples obtained from *C. subulatus* and* C. rigidus*, oxygenated Monoterpenes were the major component of all screened callistemon samples. Eucalyptol represents the major EO component in both Hydrodisillation (HD) and headspace (HS) EOs of *C. subulatus* and* C. rigidus.* In the case of *C. subulatus* HD-EO, eucalyptol (38.29%) and *α*-pinene (22.42%) representing the main EO components and were the major constituents of HS-EO, but the percentages were 59.53% and 27.96% for eucalyptol and α-pinene, respectively. In the case of *C. rigidus* HD-EO, eucalyptol (69.93%) represented the main EO component and represented the major component in *C. rigidus* HS EO, with 93.32% of the EO. From this we conclude that head space extraction technique was more efficient in isolation of higher percentage of the major EOs components from both plants.

On the other hand, the GC results for the EOS samples obtained from *P. gracilior* and* P. elongatus*, monoterpene and sesquiterpene hydrocarbons were the major component of all screened Podocarpus samples. *P. gracilior* HD-EO, *β*-caryophyllene (16.14%), germacrene D (15.66%), and *α*-pinene (12.2%) represented the main EO components, but α-pinene was the major constituent in HS-EO with a percentage of 82.01%. For the EO component of *P. elongatus*, the major component of the HD-EO was bicyclogermacrene (17.3%) and germacrene D (12.83%), whereas the HS-EO was rich in myrcene, with a percentage of 79.05%.

The percentages estimated for each chemical class varied, as in the case of oxygenated monoterpenes, the major chemical class in the samples of *Callistemon* species was 41.28 (HD), 39.62 (HS), 11.44 (HD) and 34.49 (HS) for *C. subulatus* and *C. rigidus,* respectively, whereas in the case of the *Podocarpus* species, the major chemical class of monoterpenes hydrocarbons was 18.23 (HD), 96.16 (HS), 7.16 (HD) and 97.53 (HS) for *P. gracilior* and *P. elongatus,* respectively. The HD results of the *Callistemon* and *Podocarpous* species coincided with previously published reports [36, 49, 50]. On the other hand, the observed variation in the percentages of some of the identified volatile constituents could be due to seasonal and geographical fluctuations [49].

Through the continuous search for a novel antimicrobial chemical entities and also for having safe food products free from pathogens, there is an increasing demand of active natural compounds due to their effectiveness against various pathogens and limited side effects if compared to synthetic chemicals and preservatives. So, natural products are considered as a safer option than synthetic ones. In this study, we investigated the potential of HD-EOs against *methicillin-resistant Staphylococcus aureus* (MRSA) (Gram-positive bacteria), which is one of the most common nosocomial pathogens responsible for severe mortality worldwide [51]. The antibacterial activities of the obtained EOs against coagulase-negative *Staphylococcus (CoNS)*, *Streptococcus viridans*, *Enterococcus faecalis, a*nd *Escherichia coli* were also investigated, and also their antifungal activities against *Candida albicans were tested* using disc diffusion assay and MIC. The results revealed that the hydrodistillation essential oils extracted from *P. elongatus, P. gracilior* and *C. subulatus* demonstrated uniform efficacy against MRSA, as evidenced by a zone of inhibition measuring 15 mm. In the case of the tested HD-EOs activities against Gram-positive bacteria (*E. faecalis, CoNS,* and* S. viridans*) and Gram-negative (*E coli*), the HD-EOs of *C. subulatus* and *C. rigidus* exhibit robust efficacy, resulting in a zone of inhibition measuring 20 mm, whereas the HD-EOs of *P. elongatus, P. gracilior* were active only against *CoNS*. Regarding the efficacy against *CoNS*, the HD-EOs of *C. subulatus* and *P. elongatus* displayed activity, with zones of inhibition measuring 10 mm, whereas the HD-EOs. *C. rigidus* and *P. gracilior* showed no discernible activity (Supplementary Figure S5). Minimal inhibition zone was observed against *E. faecalis*, specifically with the HD-EOs. of *C. subulatus* and *C. rigidus*, whereas no activity was observed with the HD-EOs of *P. gracilior* and *P. elongatus*. In the case of *S. viridans*, both *C. rigidus* and *C. subulatus* exhibited activity, with zones of inhibition measuring 16 mm, whereas the HD-EOs of *P. gracilior* and *P. elongatus* displayed no observed inhibitory effects. Table 3 illustrates the calculated MIC of the tested samples against different clinical isolates revealing the higher potency of HD-EOs against *S. viridans* with MIC 0.88 and 1.75 µl/ml of *C. rigidus* and *C. subulatus* respectively and the higher potency of *P. elongatus* HD-EOs against *CoNS* with MIC 0.88 µl/ml. The observed variations in the activities are generally attributed to differences in the peptidoglycan layer located outside the outer cell membrane of the bacteria. On the other hand, the outer membrane of Gram-negative bacteria consists of phospholipids (double layer) connected with lipopolysaccharides (inner membrane). Therefore, large hydrophobic molecules, such as HD-EOs components, can pass through the double membrane [52]. The potent activity of HD-EO *P. elongatus* is due to the high percentage of nonoxygenated sesquiterpene hydrocarbons represented by germacrene D, which reportedly has antimicrobial activity in the skimmed literature [53], is closely related to the composition of the oil sample. However, the HD-EO of *C. rigidus* displays potent antimicrobial activity in *E. coli* and *S. viridans* because of the high percentage of eucalyptol (oxygenated monoterpene), which has been extensively reported to have high antimicrobial activity [54]. Finally, the synergistic or antagonistic interaction between the HD-EO components could play a vital role in the antimicrobial activity of the tested oil samples.

**Table 3:** Minimum inhibitory concentrations (MICs) of the extracted EOs against different clinically isolated pathogens using microdilution broth assay

Organism	MIC of HD-EO µl/ml			
	**P. gracilior**	**P. elongatus**	**C. rigidus**	**C. subulatus**
*MRSA*	14.00	7.00	112.50	28.10
*E. faecalis*	-	225	112.50	28.10
*CoNS*	28.1	0.88	28.10	7.00
*E. coli*	-	-	0.88	7.00
*S. viridans*	-	-	0.88	1.75
*C. albicans*	-	-	-	-

**Fig. 1:**
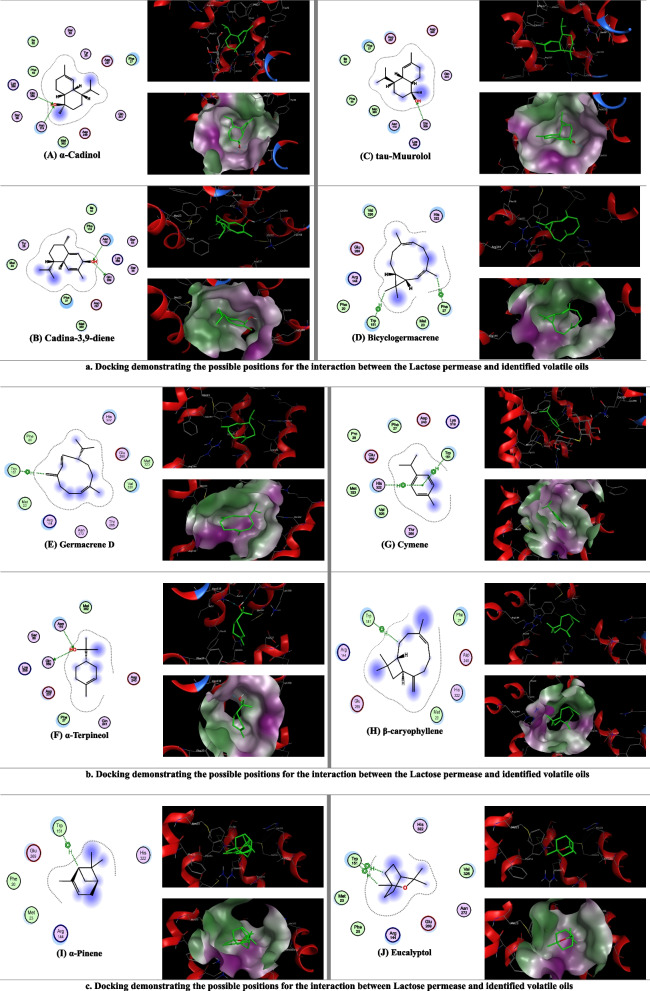
**a** Docking demonstrating the possible positions for the interaction between the Lactose permease and identified volatile oils. **b** Docking demonstrating the possible positions for the interaction between the Lactose permease and identified volatile oils. **c** Docking demonstrating the possible positions for the interaction between Lactose permease and identified volatile oils

Cellular transport proteins are integrated membrane proteins that are classified into two major classes. The first class comprises major facilitator transporters (MFS) [55], including lactose permease protein isolated from *E. coli* [56], which is responsible for the creation of a solute concentration gradient from the energy of the proton gradient. The second class of transport proteins comprises ATPases and ABC transporters, which utilize the energy (*Pi*) released from ATP to cause solute assembly or efflux. LacY is responsible for the reactions of translocation triggered by the system of galactoside transport in *E.* coli as one of the oligosaccharide/proton exchanges of the MFS transporters [56]. The inhibition of the efflux pump is considered one of the strategies that is used efficiently in the control of bacterial resistance. Efflux pump inhibitors can work through several mechanisms, such as energy uncoupling, direct binding, inhibition of gene expression, iron chelation, and changes in membrane fluidity, and these inhibitors could constitute the core of antibiotic analogs [57]. Therefore, in this study, direct binding to the efflux protein was assumed to be one of the mechanisms responsible for the observed antibacterial effect. Figure 1 illustrates all the interactions with the amino acids present at the docking site of lactose permease transport protein for ten of the identified volatile oils from all the examined plants. This figure shows the orientation of the examined ligands inside the binding site, which allows the hydroxy group of the examined oxygenated monoterpenes (α-cadinol, tau-muurolol, cadina-3,9-diene, and α-terpineol and eucalyptol) to extend into the hydrophilic cavity and form hydrogen bonds with the side chain amino acid Gln 359 Asn 119 and Trp 151, thereby increasing the stability of the ligand/lactose permease complex. For the examined nonoxygenated hydrocarbons (bicyclogermacrene, germacrene D, cymene,* β*-caryophyllene, and α-pinene), the results suggest that van der Waals, π-allyl and hydrophobic interactions with Trp151, and His322 amino acids also help stabilize the ligand/lactose permease complexes. Therefore, an interaction with a binding energy range of − 5.53 to -4.05 kcal/mol was observed in volatile oils, with significant binding affinity and stability (Table 4).

**Table 4:** Binding energies (∆G) of the identified volatile constituents using hydro-distillation (HD), and headspace (HS), within the active sites of NorA efflux pump through molecular docking and the data expressed in kcal/mol

No	Compound	∆G (Kcal/mol)
1.	α-Cadinol	-5.53
2.	Cadina-3,9-diene	-5.18
3.	tau-Muurolol	-4.98
4.	Bicyclogermacrene	-4.81
5.	Germacrene D	-4.71
6.	α-Terpineol	-4.50
7.	Cymene	-4.41
8.	β-caryophyllene	-4.32
9.	α-Pinene	-4.06
10.	Eucalyptol	-4.05

## Conclusion

The quantitative GC analysis of the EOs obtained from *C. subulatus, C. rigidus, P. gracilior,* and *P. elongatus* leaves revealed that headspace extraction is more efficient than conventional hydrodistillation in extraction of the major volatile component from each plant. *α*-Eucalyptol is considered the major discriminating marker of Callistemon species, whereas caryophyllene, germacrene and myrcene are considered the major discriminating marker of Podocarpous species. The antimicrobial investigation of the tested HD-EOs of the four plants showed higher potential against both G-ve and G + ve clinical isolates, more specifically against *MRSA*. The obtained results are due to the synergism that occurs based on the odoriferous mixtures of oils, which is one of the possible explanation for the obtained efficacy. Another explanation for the efficacy was delivered through *in-silico* docking screening of the major identified EOs for the first time against Lactose permease transporter and the results revealed moderate binding affinity of the recognized compounds with the target efflux pump enzyme opening the gate for future chemical modification of the pure compounds to improve their binding affinity and increase their antibacterial effect. Overall, this comprehensive study not only provides valuable insights into the diverse EOs chemical compositions and their antimicrobial activities, but also lays the foundation for potential applications in combating bacterial resistance through interactions with membrane transport proteins. These findings contribute to the understanding the mechanism of natural EOs as antimicrobial agents and offer prospects for the development of novel chemical entities.

## Supplementary Information

Supplementary Material 1

## Data Availability

All data generated or analyzed during this study are included in the manuscript and/or its supplementary information files.

## Abbreviations

Eos: Essential oils
GC/MS: Gas chromatography‒mass spectrometry
HD: Hydrodistillation
HS: Head-space solid phase
MH: Muller Hinton
MIC: Minimum inhibitory concentration
ZOI: Zone of inhibition
Lacy: Lactose permease transporter
MRSA: Methicillin-resistant *Staphylococcus aureus*
CoNS: Coagulase-negative Staphylococcus
