# Supplementary material for: Comparative chemical and antimicrobial evaluation of the essential oils from Callistemon and podocarpus species supported by in-silico molecular simulations against bacterial LacY protease
Source: BMC Complement Med Ther. 2025 Apr 21;25:144. doi: 10.1186/s12906-025-04826-w (PMC12012950; doi:10.1186/s12906-025-04826-w)
Supplement: Supplementary file 1 — Supplementary Material 1 [file 12906_2025_4826_MOESM1_ESM.docx]

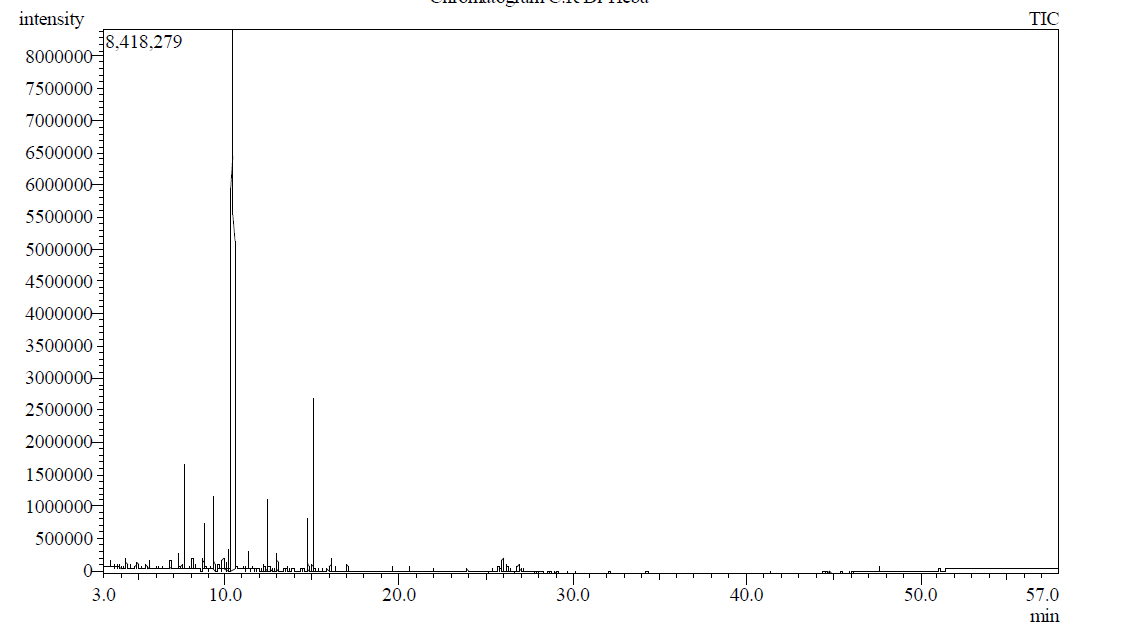


**Figure S_1_ A: Total ion chromatogram for essential oil obtained by Hydro-distillation chromatogram of *Callistemon rigidus***


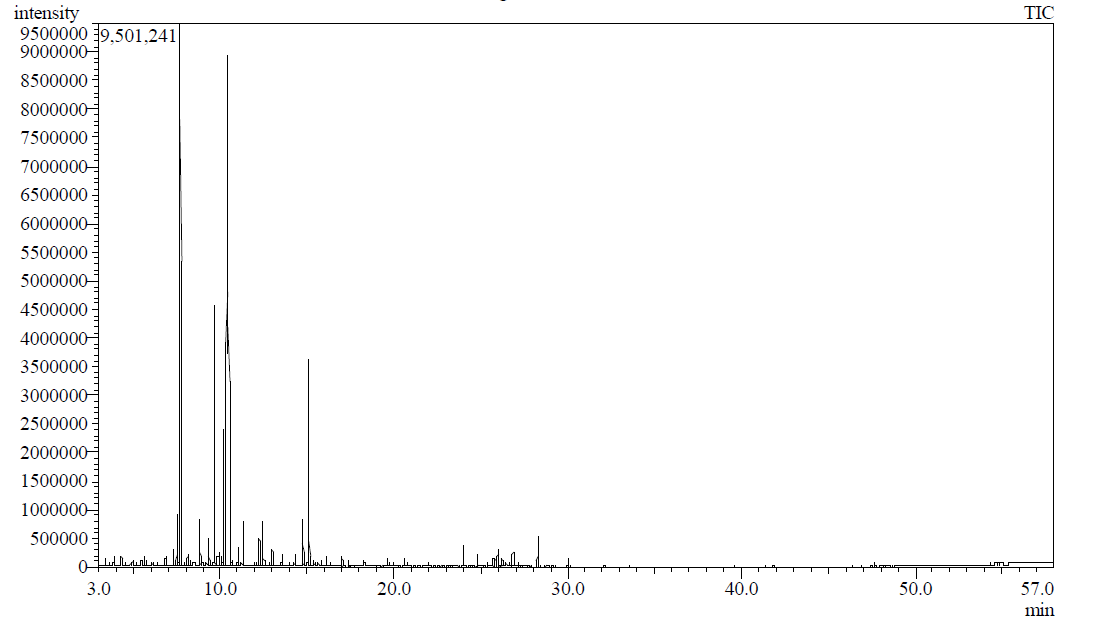


**Figure S_1_ B: Total ion chromatogram for essential oil obtained by Hydro-distillation chromatogram of *Callistemon subulatus***


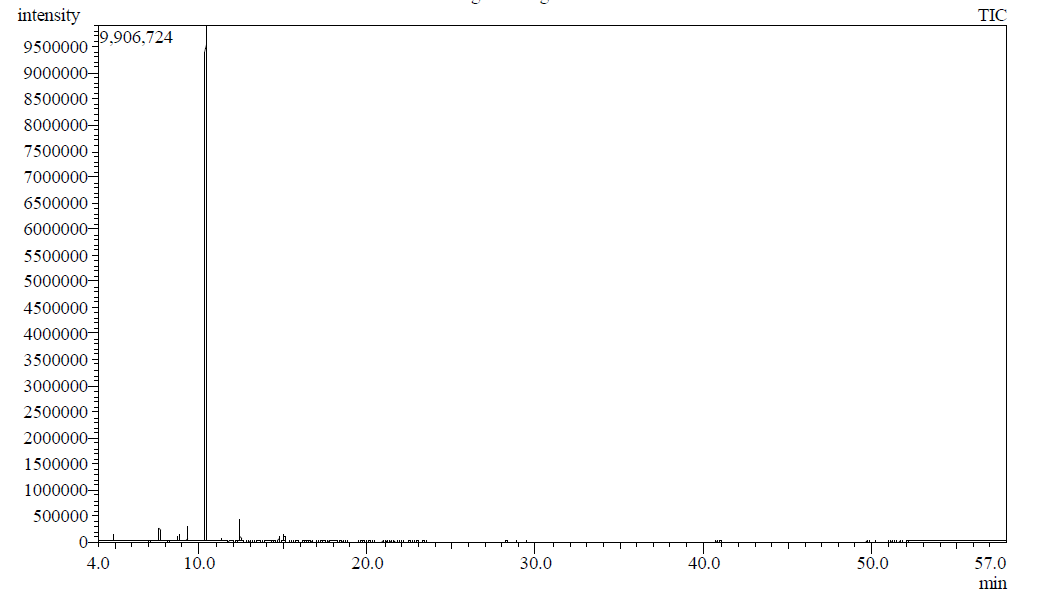


**Figure S_2_ A: Total ion chromatogram for essential oil obtained by Head-space chromatogram of *Callistemon rigidus***


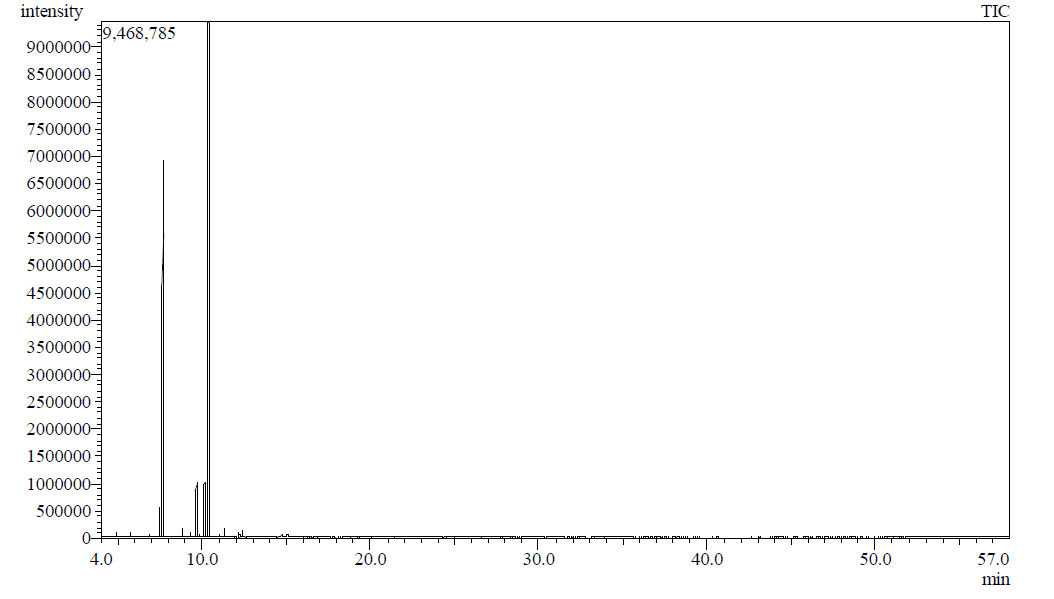


**Figure S_2_ B: Total ion chromatogram for essential oil obtained by Head-space chromatogram of *Callistemon subulatus***


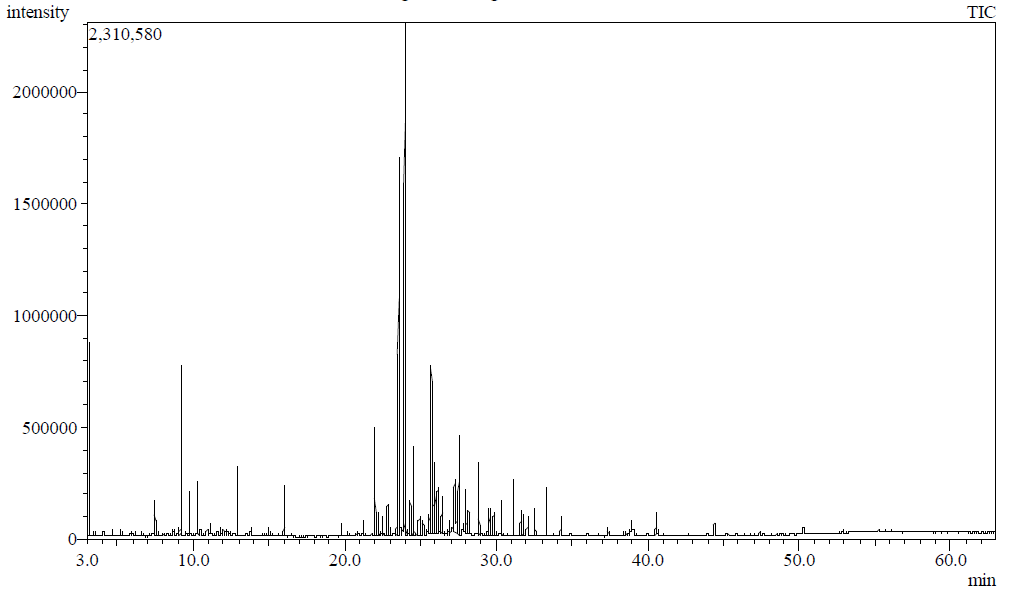


**Figure S_3_ A: Total ion chromatogram for essential oil obtained by Hydro-distillation chromatogram of *Podocarpus elongatus***


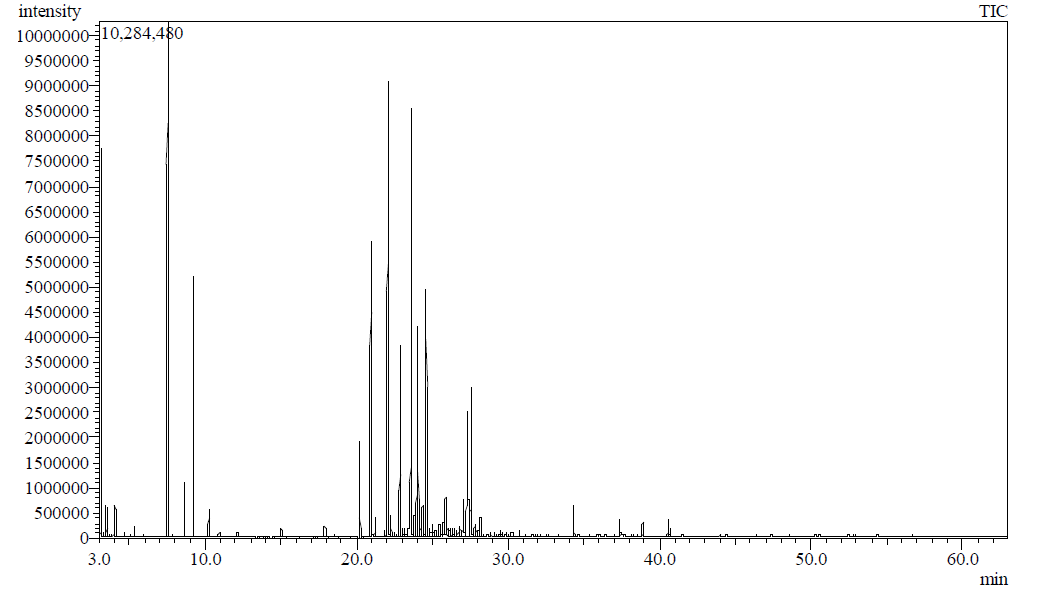


**Figure S_3_ B: Total ion chromatogram for essential oil obtained by Hydro-distillation chromatogram of *Podocarpus gracilior***


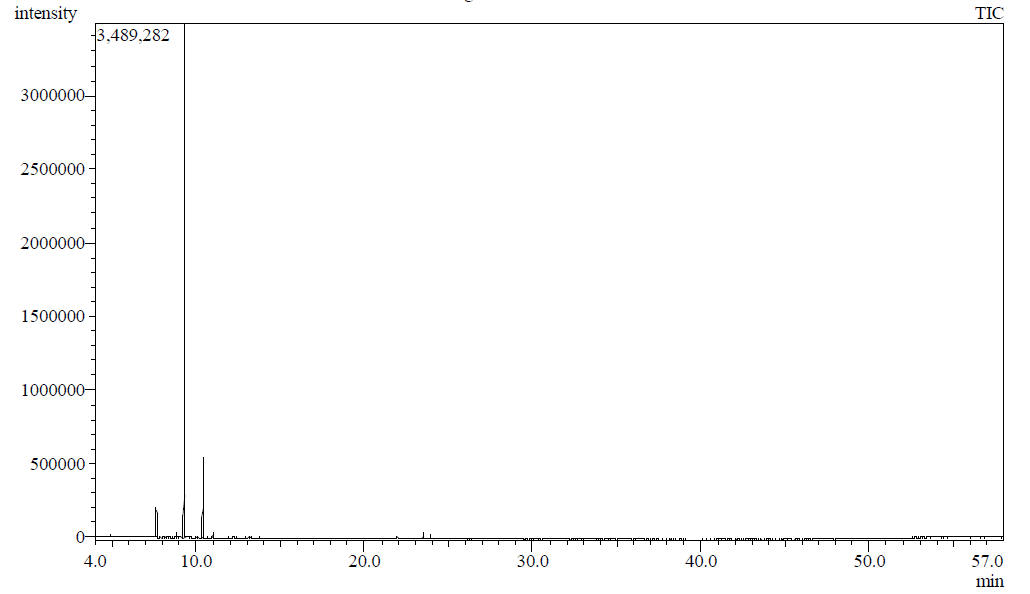


**Figure S_4_ A: Total ion chromatogram for essential oil obtained by Head-space chromatogram of *Podocarpus elongatus***


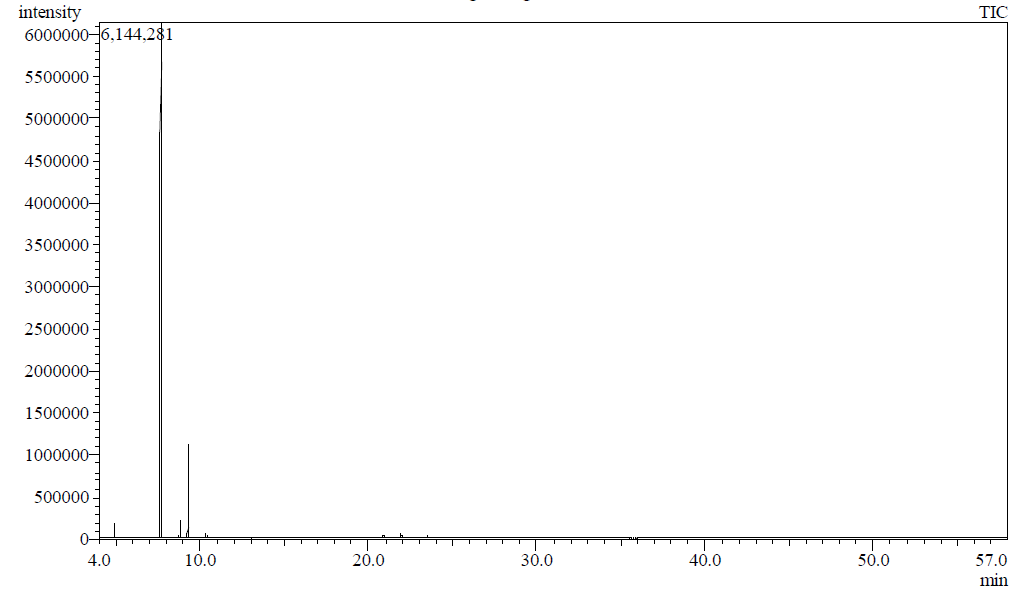


**Figure S_4_ B: Total ion chromatogram for essential oil obtained by Head-space chromatogram of *Podocarpus gracilior***


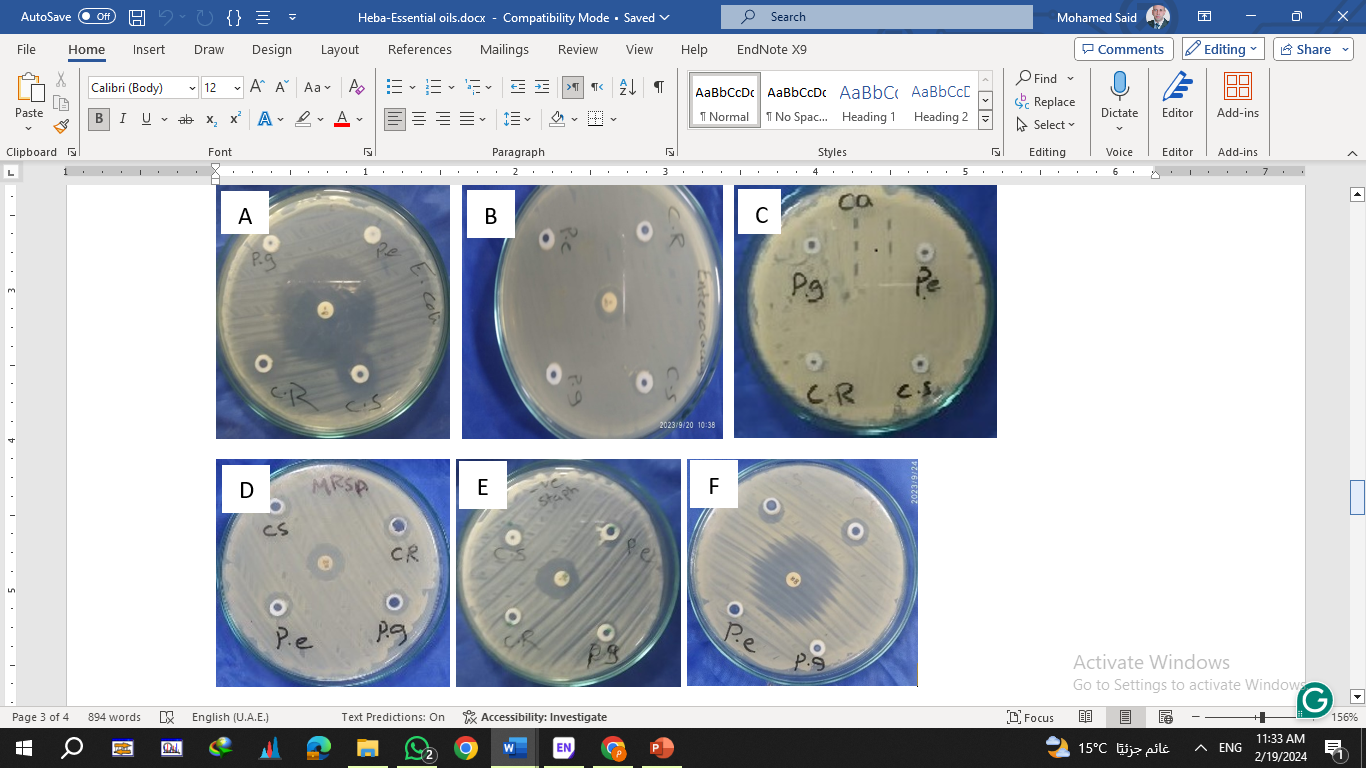


**Figure S_5_:** Results of Agar Well Diffusion Assay**. A:** The activity of EO and ciprofloxacin on *E.coli*. **B:** The activity of EO and ciprofloxacin on *E.faecalis***. C:** The activity of the oils on *C.albicans.* **D:** The activity of the oils and doxycycline on *MRSA***.E:** The activity of oils on *CoNS.* **F:** The activity of EO on *S.viridans***.**
